# What do zebra finches learn besides singing? Systematic mapping of the literature and presentation of an efficient associative learning test

**DOI:** 10.1007/s10071-023-01795-w

**Published:** 2023-06-10

**Authors:** ChuChu Lu, Agnieszka Gudowska, Joanna Rutkowska

**Affiliations:** 1 Institute of Environmental Sciences, Faculty of Biology, Jagiellonian University, Kraków, Poland; 2 Institute of Systematics and Evolution of Animals, Polish Academy of Sciences, Kraków, Poland

**Keywords:** Cognition, Memory, Associative learning, *Taeniopygia guttata*, Songbird

## Abstract

The process of learning in birds has been extensively studied, with a focus on species such as pigeons, parrots, chickens, and crows. In recent years, the zebra finch has emerged as a model species in avian cognition, particularly in song learning. However, other cognitive domains such as spatial memory and associative learning could also be critical to fitness and survival, particularly during the intensive juvenile period. In this systematic review, we provide an overview of cognitive studies on zebra finches, with a focus on domains other than song learning. Our findings indicate that spatial, associative, and social learning are the most frequently studied domains, while motoric learning and inhibitory control have been examined less frequently over 30 years of research. All of the 60 studies included in this review were conducted on captive birds, limiting the generalizability of the findings to wild populations. Moreover, only two of the studies were conducted on juveniles, highlighting the need for more research on this critical period of learning. To address this research gap, we propose a high-throughput method for testing associative learning performance in a large number of both juvenile and adult zebra finches. Our results demonstrate that learning can occur in both age groups, thus encouraging researchers to also perform cognitive tests on juveniles. We also note the heterogeneity of methodologies, protocols, and subject exclusion criteria applied by different researchers, which makes it difficult to compare results across studies. Therefore, we call for better communication among researchers to develop standardised methodologies for studying each cognitive domain at different life stages and also in their natural conditions.

**Supplementary Information:**

The online version contains supplementary material available at 10.1007/s10071-023-01795-w.

## Introduction

The juvenile period is when the most intensive learning takes place (e.g. Brust et al. 2014; Campbell et al. 2017). The process of learning is subjected to constraints and trade-offs, the outcome of which affects the entire life of an animal (e.g. Buchanan et al. 2013). Thus, in addition to adulthood, anyone interested in studying learning should also consider the juvenile period. In behavioural and neurological studies, song learning by juvenile birds has indeed gained much attention (Böhner 1983; Carouso-Peck et al. 2020; Funabiki and Konishi 2003; Menyhart et al. 2015). However, there are many different cognitive domains beyond song learning. For example, social learning, spatial orientation, and memory could be important for foraging success, food-caching behaviour, and migration (Collet et al. 2021; Healy and Hurly 2004; Shettleworth 2010; Van Leeuwen et al. 2021), while inhibitory control, associative, social, and motoric learning may be important for both foraging (Chantal et al. 2016; Osbrink et al. 2021) and reproductive success (Guillette et al. 2016).

Avian cognitive studies have long been focused on species such as pigeons (*Columba livia*), parrots (Psittacinae), chickens (*Gallus gallus*), and crows (Corvidae) (Emery 2006). Nevertheless, the zebra finch (*Taeniopygia guttata)* has more recently been regarded as a model species for the investigation of various cognitive abilities among songbirds, with a particular emphasis on song learning (Healy et al. 2010; Riebel 2009). Since developing and maintaining brain functionality for all cognitive abilities can be costly, trade-offs between different domains may be expected. For instance, song complexity was shown to be negatively related to inhibitory control and spatial learning (Anderson et al. 2017; Boogert et al. 2011; Sewall et al. 2013). On the other hand, a positive correlation between song complexity and learning proficiency has also been found (Boogert et al. 2008). In addition, some studies suggest the potential for general cognitive performance or intelligence factors across several non-song learning domains (Ashton et al. 2018; Shaw et al. 2015). Relationships between separate learning domains can only be studied if more than one is assessed in the same individual. In search of methodologies suitable for studying cognitive domains other than song learning in zebra finches, we have created a catalogue of the approaches that have been implemented so far.

**Fig. 1:**
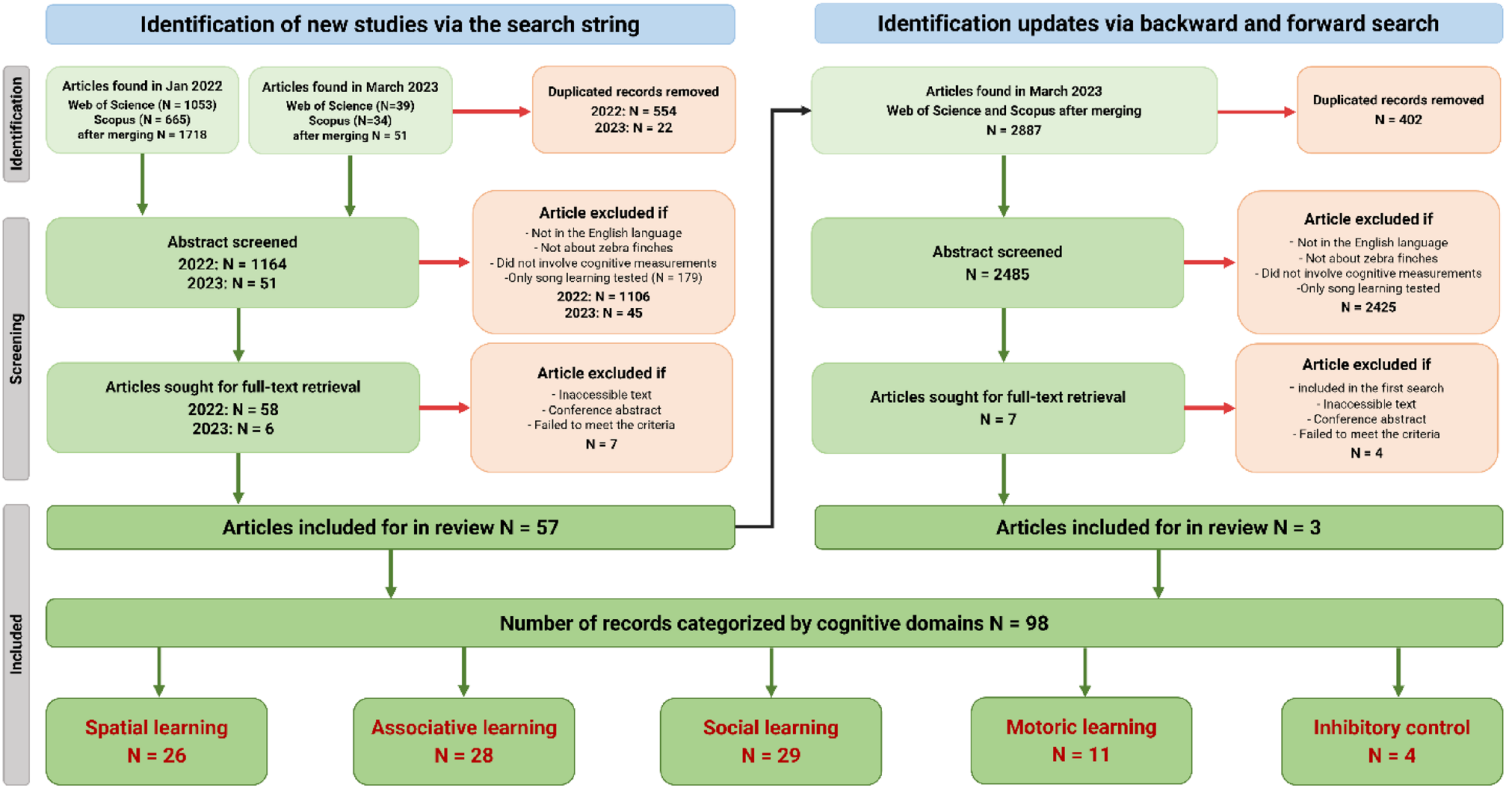
PRISMA diagram illustrating the procedures implemented during the literature search to systematically map the methodologies used to study the cognitive domains of zebra finches besides song learning. Sample sizes in the black text denote the number of articles included at each step, while the text in red indicates the number of records (data entry of cognitive tests). (colour figure online)

In this paper, we use a transparent and systematic mapping process to identify the studies concerning cognitive tests in zebra finches (Fig. 1). Our search strings were developed based on the broader categorisation of cognitive abilities presented by Griffin et al. (2015). It allowed us to identify five non-song learning cognitive domains of zebra finch research, including motoric, associative, social, spatial, and inhibitory control (see detailed description in Table 1). We provide a quantitative overview of the learning test methodologies used in those domains and emphasise the main study factors, life stages, captivity status, sex of the subjects, sample sizes, and research effort. It is also important to note that certain learning outcomes may be indirectly influenced by the test methodology, motivation, learning styles, and strategies that are more broadly known under the concept of animal personality. There is a growing interest in the relationship between learning and personality (Carere and Locurto 2011; Dougherty and Guillette 2018), as well as concerns about the potential biases in the interpretation of cognitive test results caused by the individual variation in their activeness and participation rate (Martina et al. 2021; Shaw and Schmelz 2017). Thus, in our systematic map, we also assess the participation of subjects in the tests to shed light on the possible influence of this variable on the outcomes of those tests. In addition, we acknowledge and bring attention to the potential ecological relevance of the emerging “problem-solving” studies which are not included in the analyses of this review but considered in the discussion.

**Table 1:** Summary of the categorisation of studies according to the cognitive domains. Criteria used for assigning each test to a particular domain and a list of the learning tests used in the publications identified by our search

Cognitive domain	Description of the domains and criteria used for the assignment of tests	List of learning tests and references
Motoric learning	The lid removal task is a common feature of all motoric learning studies. The birds are presented with wells that are covered by lids, which must be lifted and removed to access the food reward inside. Studies categorised within this domain explicitly evaluate a bird’s performance in motoric learning tests as a response variable. While tests of other domains may modify this setup to establish a baseline level of skill before conducting subsequent cognitive assessments, they do not analyse motoric training independently (e.g. associative learning with coloured lids)	Lid removal: Boogert et al. 2008; Campbell et al. 2017; Crino et al. 2014; Daria et al. 2022; Goodchild et al. 2021; Grindstaff et al. 2012; Howell et al. 2019, 2020; Osbrink et al. 2021; Slevin et al. 2020; Templeton et al. 2014
Associative learning	The association between colour or pattern and reward is formed during a learning phase and is subsequently assessed by giving focal birds a choice. Commonly used in the learning and testing phase are panels with lid-covering wells. Thus, the subject's ability to remove the lids is required, either by flipping with the beak or pulling the strings attached to the lids. Associative learning may also be tested by choice chambers or multiple corridors in which rewarded and non-rewarded feeders are placed. Such an approach would not rely on motoric ability/learning to remove the lids. Aversive food association can be another technique used to test the learning of an aversive cue	Lid removal: Brust et al. 2013, 2014; Danner et al. 2021; Howell et al. 2019; Jha and Kumar 2017; Kriengwatana et al. 2015; Lambert et al. 2021, 2022; Osbrink et al. 2021; Patel et al. 1997; Rojas-Ferrer and Morand-Ferron 2020; Sanford and Clayton 2008; Slevin et al. 2020; discrimination task: Osbrink et al. 2021; Rojas-Ferrer and Morand-Ferron 2020; Watanabe et al. 2008, 2011; choice chamber: Gibelli and Dubois 2017; choice corridor: Chantal et al. 2016; Fisher et al. 2006; Lu et al. 2022; curtain compartment: Bonaparte et al. 2011; food aversion: Tokarev et al. 2011
Social learning	The fundamental components of social learning tests are the observers and demonstrators. Observer birds are required to watch and learn the behaviour shown by the demonstrator birds. Behaviours demonstrated by live or video-recorded birds include nest-building and foraging using specific coloured materials and lid removal. A computer-controlled feeding apparatus may also be used to assess the learning between partners through foraging scans	Demonstration of foraging: (Benskin et al. 2002; Camacho-Alpízar et al. 2021; Guillette et al. 2014, 2022; Guillette and Healy 2014, 2017; Katz and Lachlan 2003; Larose and Dubois 2011; Mora and Forstmeier 2014; Osbrink et al. 2021; Riebel et al. 2012; Templeton et al. 2017; Van Leeuwen et al. 2021); demonstration of nest-building: Breen et al. 2019; Guillette et al. 2016; Guillette and Healy 2019; Lid removal: Easter et al. 2022; Farine et al. 2015; foraging scan: Beauchamp and Kacelnik 1991
Spatial learning	Spatial learning tests are used to assess a subject’s ability to recognise the location of the correct feeder based on environmental cues (e.g. surrounding and distant objects). Apparatuses such as 4-arm and T-maze are designed to assess the ability to navigate through the corridors. Lid-covered wells or dotted/striped feeders may also be used to assess the ability to locate the reward based on the spatial orientation of the patterns on a smaller scale	4-arm and T-maze: Bailey et al. 2009, 2013, 2017; Kosarussavadi et al. 2017; Rensel et al. 2013; Spence et al. 2009; spatial orientation in an aviary/cage: Bischof et al. 2006; Daria et al. 2022; Goodchild et al. 2021; Kriengwatana et al. 2015; Mayer et al. 2010; Templeton et al. 2014; Watanabe et al. 2011; Watanabe & Bischof 2004; lid removal: Campbell et al. 2017; Hodgson et al. 2007; Jha and and Kumar 2017; Kriengwatana et al. 2015; Oberlander et al. 2004; Osbrink et al. 2021; Patel et al. 1997; Swaddle et al. 2017
Inhibitory control	Inhibitory control requires a subject to restrain itself from certain actions and to find a way around to solve the task. In the detour task, a food reward is placed inside an opaque cylindrical tube that can only be reached by the side opening. It can be used to assess a bird’s ability to learn to avoid pecking directly on the tube and retrieve the reward from the side	Detour task: Danner et al. 2021; Daria et al. 2022; Osbrink et al. 2021; Swaddle et al. 2017

The current methodologies applied in various cognitive domains include extensive training and learning procedures that require care and observation of the birds by the experimenters. Therefore, in addition to the catalogue, we also present a more efficient methodology to test the associative learning performance in both juvenile and adult zebra finches. We were able to achieve high-throughput testing of a large number of individuals, which was applied within the short period of the juvenile developmental stage and showed that those individuals were able to learn and complete the task.

## Methods

### Systematic map

This review is based on the published articles on cognitive research in zebra finches with an emphasis on learning domains that are not related to song learning, which is already well documented (e.g. Riebel 2009). We aimed to quantitatively assess the methodologies currently used in the tests of various learning domains. A systematic mapping protocol was used to find the published studies (Fig. 1). Search for the relevant peer-reviewed literature was carried out in Scopus and Web of Science databases on 18 January 2022 and updated on 23 March 2023. Sets of keywords were used in the following search strings using Boolean logic, Scopus: (“zebra finch*” OR “Taeniopygia guttata” OR “T. guttata”) AND (“cogniti*” OR (“associative” W/2 “learning”) OR (“spatial” W/2 “learning”) OR (“social” W/2 “learning”) OR (“motor*” W/2 “learning”) OR “problem*solving” OR memor* OR “operant” OR “conditioning” OR “recognition”); Web of Science: (“zebra finch*” OR “Taeniopygia guttata” OR “T. guttata”) AND (“cogniti*” OR (“associative” NEAR/2 “learning”) OR (“spatial” NEAR/2 “learning”) OR (“social” NEAR/2 “learning”) OR (“motor*” NEAR/2 “learning”) OR “problem*solving” OR memor* OR “operant” OR “conditioning” OR “recognition”).

The number of articles identified using the above search method was 699 from Scopus and 1092 from Web of Science (Fig. 1). After merging the duplicates from the two databases using the Zotero reference manager (https://www.zotero.org/), the total number of articles considered for abstract screening was 1215. All unique records were uploaded to Rayyan (https://rayyan.qcri.org/) for screening based on the title, abstract, and keywords. Two researchers independently performed parallel screening of the abstracts. We excluded the articles that did not fulfil the criteria outlined in the PECO statement (Population: zebra finches, Exposure: cognitive tests, Comparator: none, Outcome: provided empirical results reflecting cognitive abilities in the domains other than song learning). Zotero was used to retrieve the full texts of the included articles (*N* = 57). On March 23rd 2023, we utilised the snowballing technique (identified all references and all citations of the papers included in the original version of the systematic review) to find records that might have remained undetected and finally included 60 articles for full-text screening. Two people screened the full texts, and eight articles were cross-checked among three people. We documented various data from each record such as cognitive domains, subject age, study factor, etc., as indicated in the supplementary materials. For each of the different cognitive tests carried out in a given paper, we provided a separate data entry (*N* = 98). Each entry was assigned to one of the learning domains based on our categorisation (Table 1). We analysed the final dataset (see supplementary materials) containing 60 articles and 98 records in R (version 3.6.2, R Core Team 2019) and visualised their content using the ggplot2 package (Wickham 2016).

### An efficient associative learning test

We used domesticated zebra finches resulting from the interbreeding of populations maintained in Kraków, Poland and Max Planck’s Department of Behavioural Neurobiology in Seewiesen, Germany. Breeding took place in a climatic chamber with a stable daytime temperature of 21 °C and 17 °C at night. Twenty pairs were placed in breeding cages (75 × 70 cm and 40 cm high) with two perches, a transparent feeder on the floor containing an ad libitum mixture of yellow millet, canary seed, red millet, and black seed (Megan, Krakow, Poland), and a drinker hanging on the wall of the cage. A mix of minced eggs and carrots was provided on alternating days. Cardboard nestboxes were attached to the inside of the cage in the top right corner, and shredded paper towels and wood wool were provided as nesting materials. Pairs were monitored daily for nest building, egg laying, incubation, and hatching progress.

**Fig. 2:**
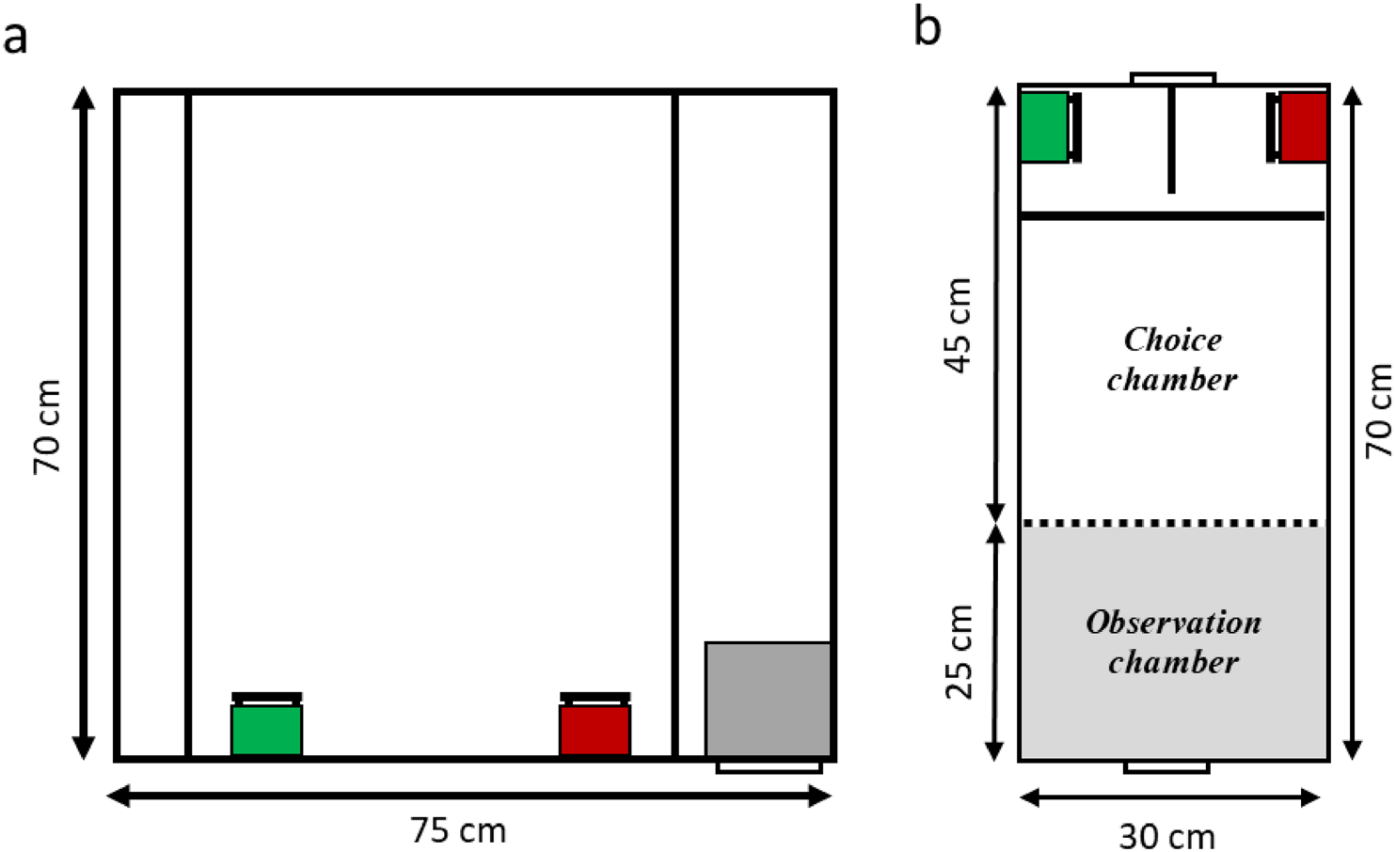
Illustration of the associative learning test setup. **a)** Home learning cage with green and red boxes representing one of the seven combinations of the colour feeders, one is rewarded, and the other is empty during the 2-week learning phase. Two vertical black lines across the cage are the perches. The grey box in the bottom right corner represents the nestbox that was hanging at the top of the cage. The black horizontal line represents the cage door. **b)** Test cage with observation chamber and choice chamber shown. The dashed line represents the removable mesh divider. The thick horizontal black line represents the perch, and the vertical line represents the divider between the two feeders. There are two doors on each side of the cage to access either chamber. The same colour combination of the feeders given during the learning phase is provided during a given test session. (colour figure online)

The learning phase was initiated at 40 days post-hatch of the youngest chick in the clutch. At that time, two opaque feeders of different colours were placed in each of the home cages for a minimum of two weeks (Fig. 2a). The colour feeders were hung on the front mesh of the home cage. Seven different colour combinations of the feeders used in the learning phase were formed using blue, burgundy, pear, and graphite. Each cage was randomly assigned a colour combination; one colour feeder was filled with seed mix and the other remained empty (Fig. 2a). The regular feeders were removed from the home cages during this phase. During the learning phase, all subjects went through three habituation sessions during which they spent 30 min alone in a cage that was the same size as the testing cage. The learning performance of two juveniles from each family was measured at 55–65 days of age, and at the same time, the performance of their parents was assessed. All learning and testing procedures were carried out in the same way as that of Lu et al (2022). All tests were conducted after overnight (1 h before lights-off at 2000 h until 2 h after lights-on at 0700 h) food deprivation to increase the motivation of the birds to perform the task.

The testing cage was divided into an observation chamber and a choice chamber by a mesh divider (Fig. 2b). The observation chamber was covered on three sides, except the meshed wall between the two chambers to provide a view of the feeders. The colour feeders were hung at the end of the choice chamber on the opposite side of the cage with a single perch placed next to the feeders. Only the reward feeder was filled with a layer of seeds while the other remained empty. There was an opaque divider between the two feeders so the bird could not see the other feeder once a choice has been made (Fig. 2b).

During the testing phase, each of the test birds was transferred to the observation chamber and allowed to calm down for 5 min while observing the feeders on the other side. The experimenter then gently lifts the meshed wall for the bird to enter the choice chamber and choose a feeder. Each was given one trial of the learning test since the lack of side and colour preference has been determined in a previous study (Lu et al. 2022). Response variables, including task participation and learning performance, were measured. Task participation reflected whether an individual participated in the test and chose a feeder (outcome: 1 = choice; 0 = no choice), regardless of whether the choice was correct or not. The outcome of the learning performance was scored as correct (1) or incorrect (0). All statistical models were built with one categorical factor of life stage (juveniles at 55–65 days old and their adult parents) and one random factor of family ID and analysed in R using the generalised linear mixed models with the package lme4 (Bates et al. 2015).

## Results and discussion

### Temporal trends and general overview of cognitive research in zebra finches

**Fig. 3:**
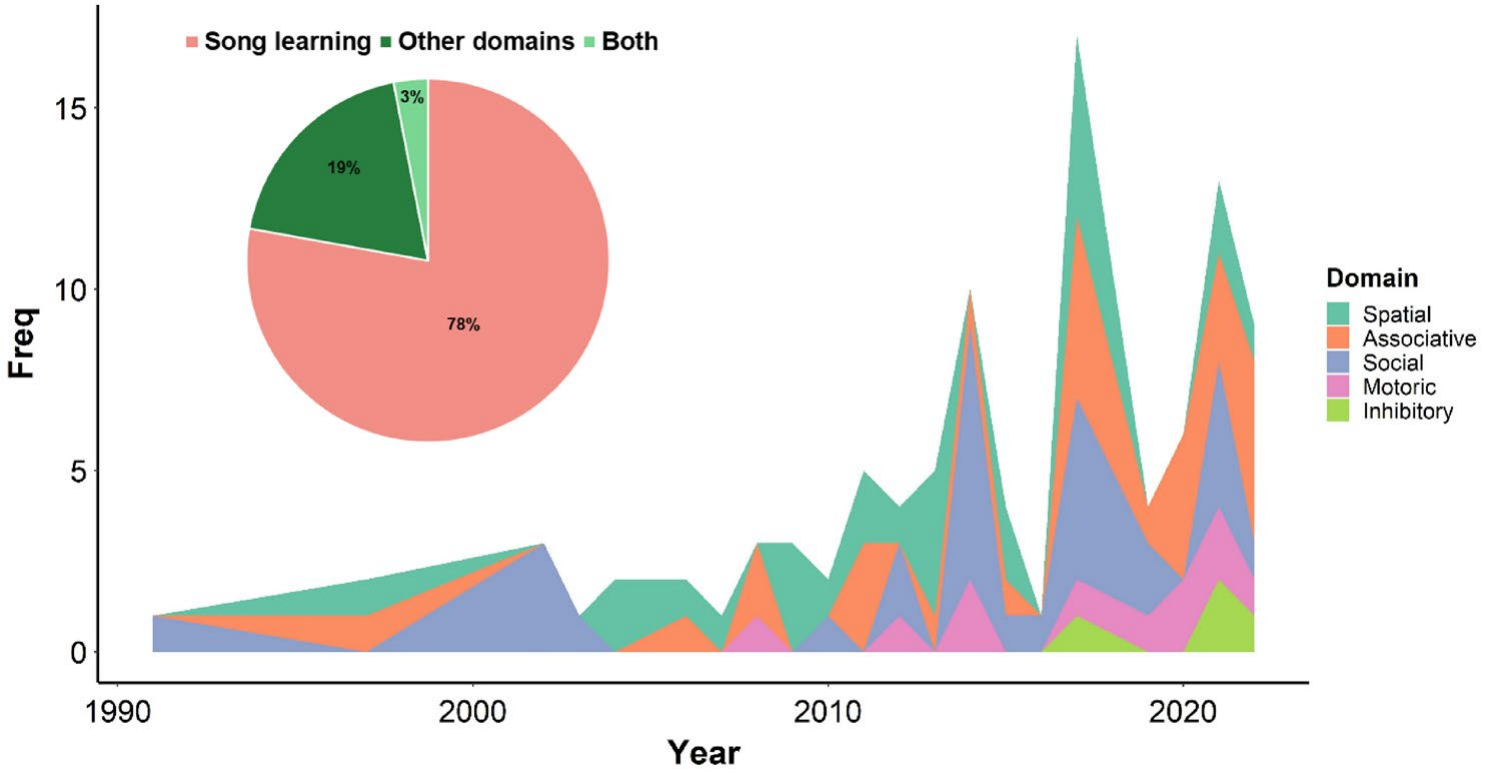
Pie chart of the percentage of empirical studies based on the first search results (January 2022) that are categorised into those studying song learning (*N* = 179), non-song learning domains (*N* = 51) and studies that tested both (*N* = 7). Timeline of the number of cognitive tests (non-song learning) described in 60 empirical articles based on the updated search results (March 2023). Different colours represent categories of learning domains (*N* = 98). (colour figure online)

In the abstract screening stage (Fig. 1), we found that 78% of the zebra finch cognitive studies were about song learning, while 19% concerned other learning domains and only 3% studied both at the same time. This shows a clear bias towards song learning among the cognitive research done on this species. We found 60 empirical studies which tested cognitive domains other than song learning. The number of such studies has notably increased over the last 20 years (Thornton and Lukas 2012, Fig. 3). Here, we discuss the five main learning domains of zebra finches that are commonly tested in relation to their foraging or nest-building abilities. Food was the primary motivator utilised for the majority of testing across all five domains. In some social learning studies, researchers relied on an individual’s intrinsic motivation to use nest-building materials. More than one learning domain could be tested in a given empirical study. The most frequently (85%) studied domains were spatial learning, associative learning, and social learning (Figs. 1, 3), while motoric learning and inhibitory control have not gained much attention (Figs. 1, 3). The motoric function is the basic ability to perform certain movements to achieve a goal and it is the most basic cognitive ability that is required to develop further learning abilities (Griffin et al. 2014). The earliest study in our data which implemented the lid-flipping compartment to test for associative learning in zebra finch was Patel et al. 1997. Later on, the “novel foraging task” introduced by Boogert et al (2008) was used to explicitly test the motoric learning ability of zebra finches based on our categorisation. This methodology has been adopted by other researchers such as Campbell et al (2017) and Goodchild et al (2021) to test the motoric function as a separate cognitive domain. However, we found that motoric tests have primarily been conducted as a training tool preceding other cognitive tests, such as associative learning (e.g. Kriengwatana et al. 2015; Swaddle et al. 2017), but were not evaluated as an independent learning test. Given the importance of motoric learning, it would be rational to analyse separately the motoric function of the birds even if it is intended to be used as the prerequisite of other learning tests.

### Main study factors

Currently, the cognitive tests of zebra finches that do not involve song learning are grouped into five domains: associative, inhibitory, motor, social, and spatial learning. We categorised the articles based on our criteria outlined in Table 1 (see also supplementary materials). For each cognitive test, we recorded the study factors of interest, including environmental factors (e.g. no-light environment or exposure to heavy metal), social factors (e.g. mating preference, characteristics of partners, familiarity, or demonstrator sex), brain (e.g. hippocampal lesions or injections), personality (e.g. the correlation between personality traits and learning ability), resources (e.g. growth impairment, nutritional stress, or dietary manipulation), physiology (e.g. microbiota, hatching asynchrony, or estradiol effect), and none (e.g. individual variation).

**Fig. 4:**
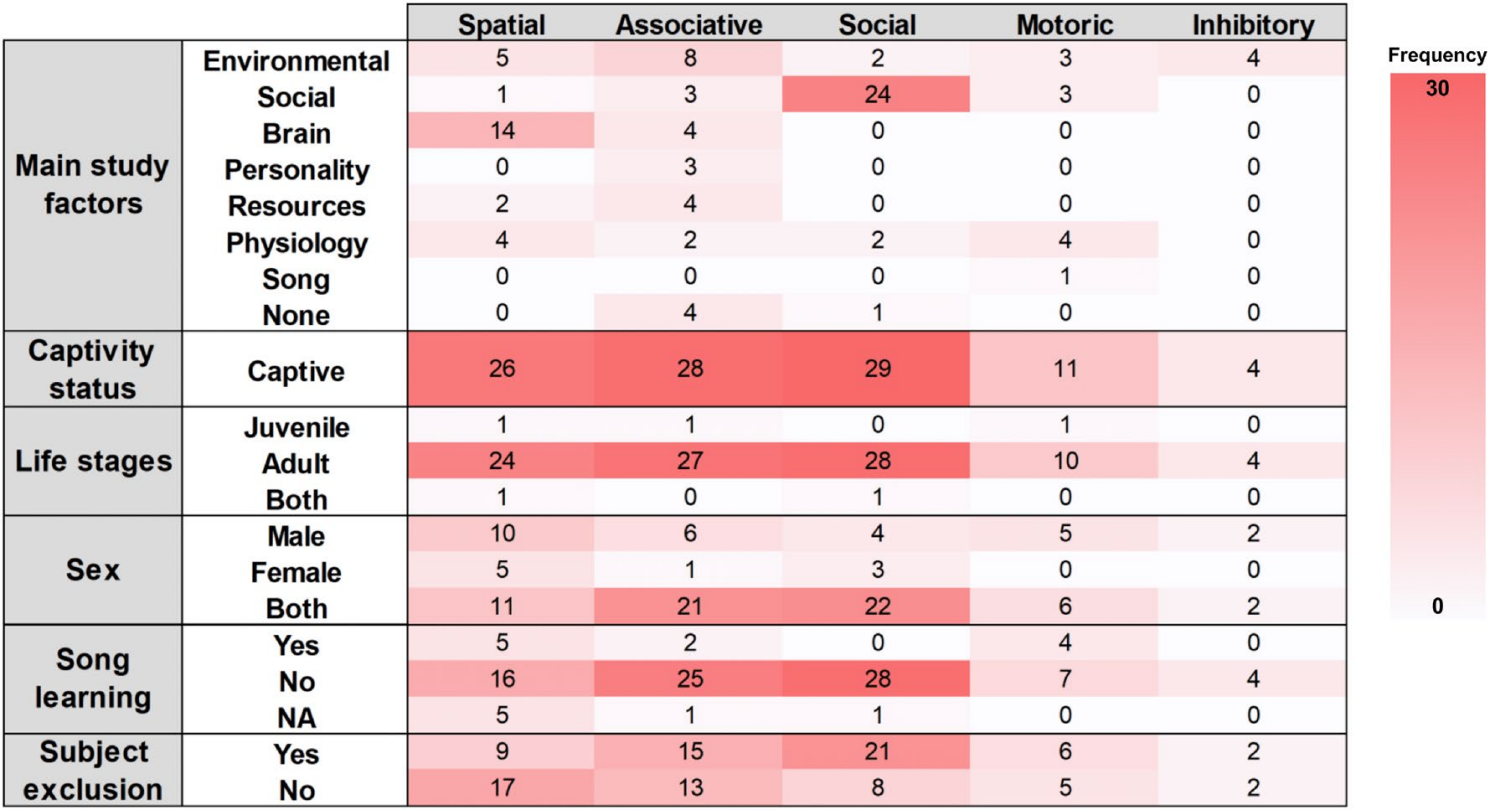
Heatmap of the number of cognitive tests described in each of the five learning domains. Different aspects of the methodologies used in cognitive research of zebra finches are represented including the main study factors, life stages, captivity status, and sex of the tested birds, whether song learning was also assessed and whether any bird was excluded from the data analyses. The colour gradient indicates the frequency of the number of records (all combinations of cognitive tests included in the articles) in this systematic map. (colour figure online)

The impact of environmental factors on various domains of learning has been widely studied, but social learning has received relatively little attention in this regard (Fig. 4). However, it is important to recognise that environmental factors, such as traffic noise, can also affect social learning by interfering with communication between individuals, just as they do with other forms of learning (Osbrink et al. 2021). Furthermore, exposure to lead has been shown to have a negative effect on the song learning ability of male zebra finches (Goodchild et al. 2021), which is closely associated with social learning in terms of brain function and regions (Bosikova et al. 2010). Thus, investigating the relationship between different environmental factors and social learning through further empirical studies could shed light on the underlying mechanisms.

The involvement of social factors, such as the sexual characteristics of the birds in the study is most often applied to social learning tests (Katz and Lachlan 2003; Van Leeuwen et al. 2021; Fig. 4). However, there is a lack of research on how social factors affect other learning domains. Only a few studies have shown that characteristics of different sexes can influence associative learning (male foraging efficiency influencing female mate choice, Chantal et al. 2016), motoric learning (female preference towards songs of males who are faster in solving a foraging task, Howell et al. 2020), and spatial learning (sex differences in spatial learning performance, Kosarussavadi et al. 2017).

Almost all (78%) studies involving brain manipulations, such as hippocampal and entopallium lesions, were done in relation to spatial learning, but rarely in the context of other learning domains (Fig. 4). Yet, negative effects of hippocampal tissue lesions have also been found on associative colour memory task performance (Patel et al. 1997). While it makes sense to investigate memory retention and spatial orientation with brain lesion techniques (Mayer and Bischof 2012; Watanabe 2001), more insights could also be gained for other learning domains. Brain manipulations are very often used in song studies (Bosikova et al. 2010), so there is the potential to investigate the effects of such treatments on cognitive domains that may be traded off or positively correlated with song learning.

Personality is another study factor, the effect of which has only been investigated in relation to a single domain, associative learning. Activity measures, such as subjects' participation and latency, have been commonly used as proxies for personality traits (Brust et al. 2013). These are important measures for all learning domains because the robustness of the cognitive test results may depend on the activity level or the willingness to participate in the test (Lu et al. 2022; Martina et al. 2021; Shaw and Schmelz 2017).

Interestingly, only associative learning tests have been conducted in relation to all types of experimental factors categorised in this map (Fig. 4). Those studies could potentially be used in a meta-analysis comparing associative learning ability with regard to the study factors. On the contrary, social learning is almost exclusively studied when the social characteristics of the individuals are manipulated (e.g. Beauchamp and Kacelnik 1991; Katz and Lachlan 2003). The only other experimental factors applied in studies of social learning were rearing conditions and physiological constraints, namely early life conditions or corticosterone manipulation (Breen et al. 2019; Farine et al. 2015; Larose and Dubois 2011; Riebel et al. 2012), though other factors and resources may also affect the efficiency of social learning as shown in other learning domains (e.g. Danner et al. 2021; Osbrink et al. 2021).

### Life stages

Most (94%) studies included were done on adult zebra finches with only two experiments which tested both the adults and juveniles (Bailey et al. 2009; Farine et al. 2015; Fig. 4). Those two studies compared the individuals of different age groups, while no study has measured the cognitive abilities of a given individual at different life stages. Such an approach would allow for the long-term assessment of the functional consequences of the environmental or physiological effects during the developmental stage compared to the environments which the subjects experience later in life (Groothuis and Taborsky 2015). Surprisingly, only two studies solely tested juvenile zebra finches, assessing their motoric, spatial, and associative learning at 40 and 60 days post-hatch (Campbell et al. 2017; Lu et al. 2022). Another study described their subjects as juvenile birds at the age of approximately 3 months old (Rojas-Ferrer and Morand-Ferron 2020). This does not follow our definition of juvenile in this map, which is less than 2 months old based on the critical period of sensory learning for zebra finches finishing at around 65 days of age (Brainard and Doupe 2000). Considering that sensory development occurs at the early stage, the fledglings must learn and develop their foraging ability, such as motoric learning, as early and efficiently as possible. Otherwise, the juveniles may have a lower chance of survival until adulthood.

### Captivity status

All studies included in our systematic review were conducted on captive animals in laboratory settings. This finding highlights a significant knowledge gap in our understanding of how the cognitive abilities of captive zebra finches may differ from those of their wild counterparts. Although laboratory environments provide controlled conditions for conducting extensive tests, it is important to acknowledge that the behavioural traits observed in captive birds may not always reflect those in nature. While some studies have suggested the comparability between captive and wild behaviour (Herborn et al. 2010), other research highlights the importance of studying birds in both conditions to fully understand the potential differences and complexities of the behavioural variations (Benson-Amram et al. 2013). Therefore, future studies on the cognitive abilities in their natural habitats are essential to better comprehend their evolutionary processes (Cole et al. 2012) as well as to transfer and apply our current knowledge obtained in the lab.

### Sexes

Studies of all cognitive domains performed experiments on both sexes (Fig. 4). Majority (75%) of the tests in associative and social learning domains were carried out on both males and females, while it is more common for spatial learning tests to focus only on males compared to other domains. Among the studies that included subjects of both sexes, few presented direct comparisons between them (Brust et al. 2013; Guillette and Healy 2014; Kosarussavadi et al. 2017; Lambert et al. 2021). Male zebra finches have been found to perform significantly better than females in a spatial learning test (Kosarussavadi et al. 2017), while others showed that male birds were better than females in a reversal learning test (Brust et al. 2013). However, in a nest-building study, there was no sex difference in learning speed (Lambert et al. 2021). Differences in cognitive abilities between the sexes may arise due to their distinct roles in reproduction and their behaviours related to courtship and breeding (Guigueno et al. 2015, 2016; Halpern 2000). Therefore, future comparisons between the cognitive abilities of the two sexes would help determine any sex-specific trade-offs between different learning domains.

### Song learning

Excluding the tests that were conducted exclusively on females (no singing behaviour in females), most (88%) of the experiments in this map did not test song learning ability in combination with other cognitive domains (but see Goodchild et al. 2021; Jha and and Kumar 2017; Templeton et al. 2014) (Fig. 4). Only spatial, associative, and motoric learning domains had experiments in which the song learning ability was also assessed on the same males that were tested (Jha and and Kumar 2017; Templeton et al. 2014). Social learning was not at all studied in parallel with song learning. This was surprising considering that song learning is a social process and the two domains have been associated with the same brain functions (Bosikova et al. 2010; Zachar et al. 2020). Overall, this knowledge gap provides one of the directions for future research, namely to investigate relationships between song learning and social learning domains.

### Subject exclusion

Exclusion of individuals from further testing or the final analyses has been observed across all learning domains, but the frequency of this practice varies greatly between different learning domains (Fig. 4). More than half of the experiments in the social learning domain involved the exclusion of the test subjects. This is mostly due to the lack of participation in the learning (observation of the demonstrator bird in this case) or the testing phase in social learning experiments. For instance, studies with nest-building tests often report individuals that failed to interact with the nest material within a time threshold (Breen et al. 2019; Guillette et al. 2016). Half of the associative learning experiments excluded some subjects due to their failure to reach certain training criteria. For example, the individuals were required to eat from at least two of the four baited wells within two minutes to pass a stage. If a bird did not pass a given stage after 60 trials, it was removed from further testing and marked as a non-solver (Howell et al. 2019). The most commonly used apparatus for associative learning is lid-covered wells, and the prerequisite of this test is the ability to perform a motoric task (Howell et al. 2019). Incorporating the prerequisite to test and analyse motoric task performance before further assessment of other learning domains may provide a better resolution of the participation and performance outcome of the latter tests.

Excluding subjects that failed to participate could potentially lead to activity bias when interpreting the results of the cognitive tests. The outcome of the test for individuals that were inactive during the tests could have been neglected due to the limitation of the set criteria or time frame (Martina et al. 2021; Shaw and Schmelz 2017). Furthermore, spatial learning greatly involves subject activity and willingness to explore the predetermined locations in the test area; however, it has not been studied with respect to personality traits (Fig. 4). Filling this gap could help to address the problems of personality biases, such as lower activity level or participation rate resulting in undetected learning performance outcome. It would also bring more insight into the relationship between personality and cognition, which has been an increasingly popular topic (Brust et al. 2013; Carere and Locurto 2011; Gibelli and Dubois 2017).

### Research effort

**Fig. 5:**
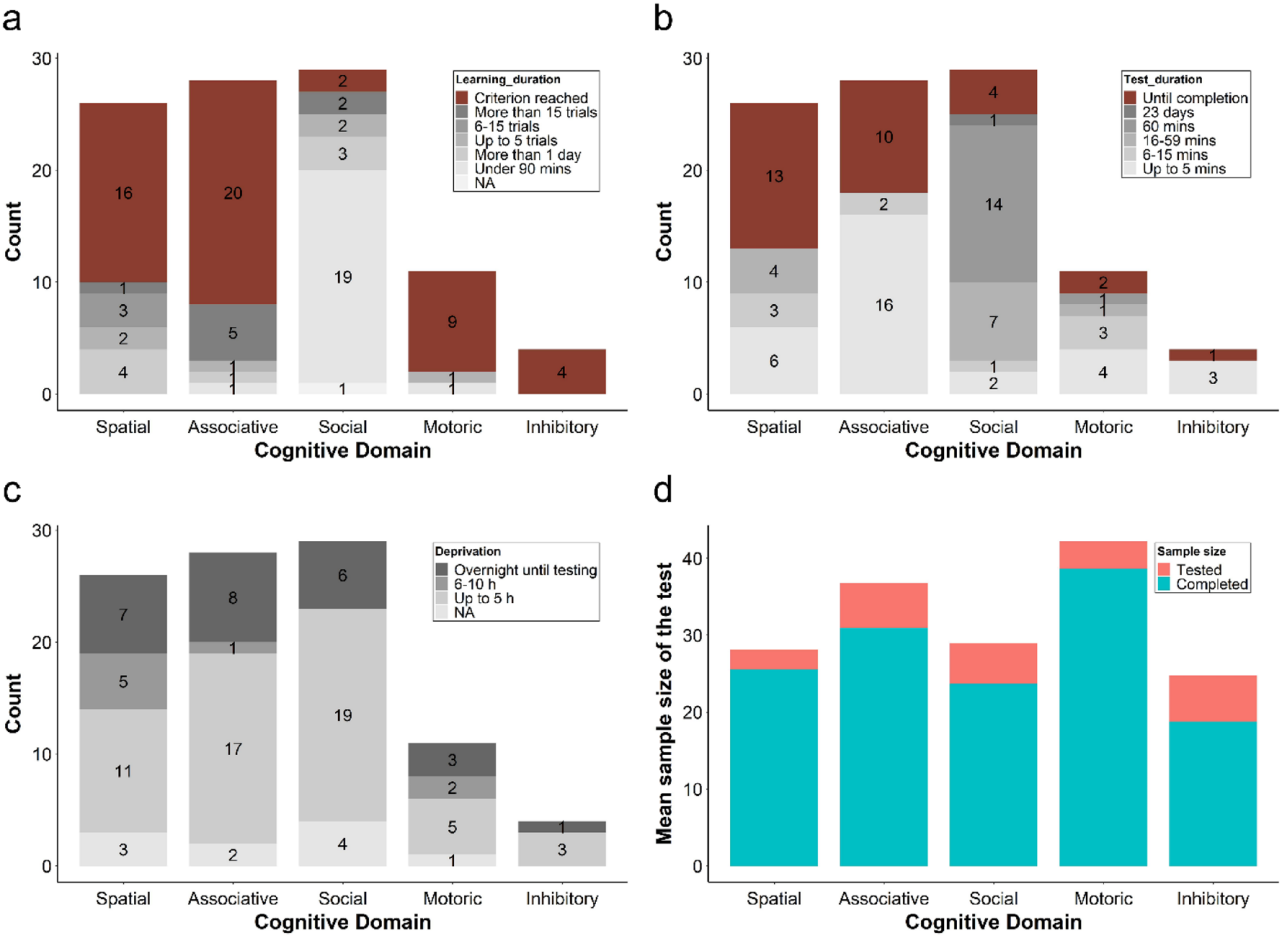
Research effort required to perform different cognitive tests described as the count of the records and the mean sample sizes tested. **a)** Number of trials and the training effort required for the subjects to advance to the testing phase. Lighter grey—less amount of effort required, darker grey—the most effort required. Brown—tests that required the subjects to reach certain criteria without a fixed time threshold. **b)** The duration of each test. Lighter grey—shorter tests which require less effort from the experimenter. Darker grey—longer test sessions. Brown—tests that last until the subjects complete a given task without a fixed time threshold. **c)** The length of the food deprivation implemented before the learning or testing phase. Lighter grey—shorter; darker grey—longer deprivation period. **d)** Mean sample sizes of the tests. Top of the Orange bar – mean of the total number of individuals tested. Height of the orang bar—mean number of excluded individuals. Blue bars within each column—number of individuals that completed the test (after exclusion of birds that did not complete or participate in the learning or testing phase). (colour figure online)

Cognitive tests often involve a learning phase where the subjects are trained to carry out certain tasks. The length of this phase varies greatly among different experiments in each of the cognitive domains (see supplementary materials). The majority (90%) of the social learning tests involved a specific period of the learning phase because the birds usually have a set observation period to watch the demonstrators. Learning phases longer than one day were commonly used in studies of nest-building, as it takes longer for the demonstrators to build and show their nests. One study on associative learning (Lu et al 2022) implemented a learning phase (similar to the one described in the current paper, Fig. 2a), which is characterised by the relatively little research effort required. Spatial learning can require learning periods of up to 20 days as multiple sessions were spread out across days. Finally, 52% of the experiments had a learning phase which required the individuals to reach set criteria before advancing to the testing phase (Fig. 5a). This type of learning phase ranged from a set number of trials per day up to several hours across many days. It is difficult to predict the actual time frame needed to carry out the learning or testing phase when either a criterion has to be reached or a test has to be run until completion (brown sections in Fig. 5a and Fig. 5b). The abovementioned procedures should be taken into careful consideration when planning experiments because of the extensive amount of research effort involved. Only one record did not implement a learning phase because the test was recorded and analysed for the entire duration (Easter et al. 2022).

After the subjects have completed the learning phase, the testing phase took place and individuals were tested for learning performance in a given domain. The duration of the test sessions varied among the different learning domains, ranging from less than 5 min up to 60 min, as well as trials that ran until a given subject has completed the task (Fig. 5b, see supplementary materials). Running the test until completion is similar to the category of criterion reached during the training phase. The same duration of testing as the learning trials is usually implemented to consistently measure the performance.

Before both the learning and testing phases, researchers often apply food deprivation procedures to increase the motivation of the subjects to complete a task. Only 12 out of 98 records did not involve or did not mention/report the deprivation procedures. The most common length of the deprivation period was up to 5 h, followed in frequency by overnight fasting until the test sessions (Fig. 5c). Carrying out food deprivation may require further effort for different tasks, such as removing the food/water or cleaning the cage floor. Catching and transferring the subjects to empty cages or the test environment can also be laborious and time-consuming.

The mean number of individuals tested in each of the experiments across all studies is 32.3 (Fig. 5d). The sample sizes vary greatly from merely 4 birds (Larose and Dubois 2011) up to 209 (Lu et al. 2022) birds tested in a single study. These are the total number of individuals initially included by the researchers. However, due to lack of participation in the tests or failure to complete the learning/training sessions, individuals from either the learning or testing phase were often excluded from further analyses (final sample sizes range between 35 and 100 percent of the tested birds, supplementary materials). The mean number of individuals eventually included in the reported results is 27.7 (Fig. 5d). Notably, this number refers to the entire study, and thus, the sample size of the studies with treatment groups would further divide this mean.

### Problem-solving tasks

In our systematic map, we have taken the classification of cognitive domains proposed by Griffin et al (2015). However, we acknowledge that some ecologically relevant cognitive abilities may not fit into these predefined categories. For example, “problem-solving” describes a particularly ambiguous task that may require a combination of different learning domains to be solved. Some studies have shown that animals can approach problem-solving tasks in different ways, such as by learning the solution through trial and error or by inferring the correct logic (Thornton et al. 2014). Moreover, some cases suggest that the task performance may not reflect cognitive abilities, but rather a motivation or other factors (van Horik and Madden 2016).

Our review included two zebra finch problem-solving studies but only the parts of the tests involving lid flipping and associative choice chambers were analysed as motoric and associative learning, respectively (Chantal et al. 2016; Templeton et al. 2014). Two other studies that focused only on problem-solving performance were not included in our review. Schmelz et al. (2015) used a test battery with three tasks to compare the problem-solving performance of three Estrildid finch species, including zebra finch. Barrett et al (2022) used the same method to investigate the link between problem-solving and personality traits in zebra finches. The tasks used in all four studies did not require extensive training or observation by the experimenters to measure the latency to solve the task by successful birds. With its relatively low research effort and significant ecological relevance, problem-solving has the potential to be a valuable area of study. While problem-solving may not typically be classified as a specific learning domain, it should be given due consideration and included in future research.

### Efficient associative learning test

**Fig. 6:**
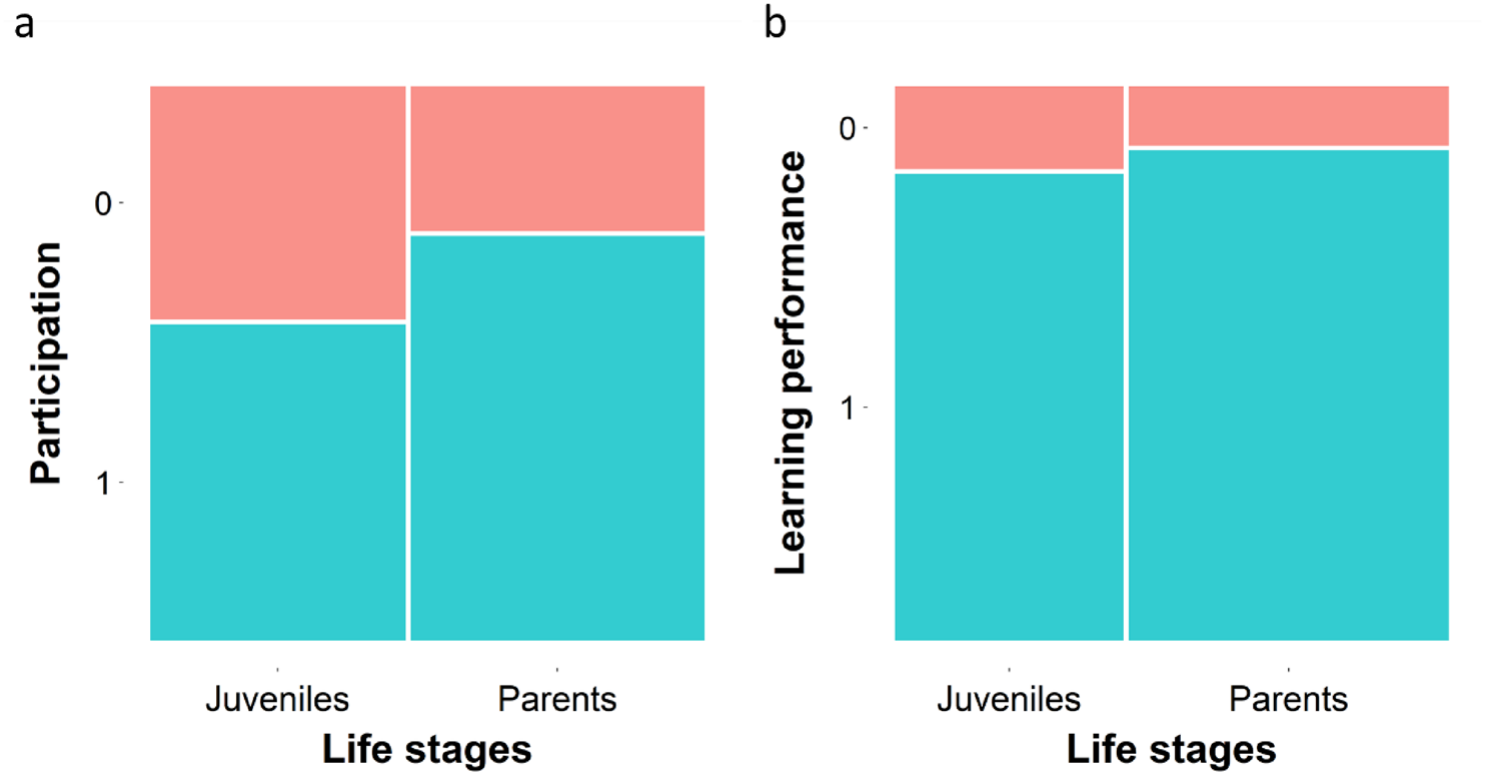
Results of the associative learning test. **a)** Participation rate of the juveniles (*N* = 36) and adults (*N* = 38). The proportion of the participants is represented by the blue panels (1 = participant, parents *N* = 28, juveniles *N* = 19) and red (0 = non-participant, parents *N* = 10, juveniles N = 14). **b)** Associative learning performance of the juveniles (*N* = 20) and adults (*N* = 28). The proportion reflecting the performance score is represented by the blue panels (1 = correct choice, parents *N* = 25, juveniles *N* = 17) and the red panels (0 = wrong choice, parents *N* = 3, juveniles *N* = 3). (colour figure online)

We tested the ability to associate specific colour feeders to the food reward in 74 juvenile and adult birds. We demonstrated that juveniles and adults were equally likely to choose one of the colour feeders within 10 min of testing (GLMM: age class: *z* = 1.58, *df* = 71, *p* = 0.11, family ID: variance = 1.289, SD = 1.135, Fig. 6a). Learning took place in both groups as their average learning performance score was significantly greater than 0.5 (one-sample *t* test: t_47_ = 7.77, *P* < 0.001, Fig. 6b), where 0.5 would indicate random choice of the colour, thus a lack of learning. There was no difference between the life stages in their associative learning performance (GLMM: *z* = 0.27, *df* = 48, *p* = 0.79, family ID: variance = 2.049, SD = 1.432, Fig. 6b).

This finding should encourage researchers to perform cognitive tests also on juveniles, with the interest of determining the effects of different experimental factors on birds at different life stages. This methodology will be useful for those who search for an efficient way of assessing learning performance in juveniles and/or of a large number of individuals. It can potentially be implemented in studies of other captive passerines, which will pave the way for further comparisons between different species. We also suggest the future possibility for inter-specific application of the various learning tests across different cognitive domains.

## Conclusions

In this review, we aimed to provide a comprehensive overview of the methodologies implemented in the field of cognitive research in zebra finches, emphasising the study factor, life stages, captivity status, sex, sample sizes, and research effort. Over the past 20 years, the number of empirical studies on non-song-related learning abilities in zebra finches has steadily increased and we have observed a higher number of experiments on spatial, associative, and social learning domains. It is important to note that all studies included in the systematic review were conducted on captive birds. While this may limit the generalisability of current findings to wild populations, studying captive animals has the advantage of the control for extraneous variables that may influence cognitive abilities. In addition, there is a significant bias towards testing subjects at the adult stage (95%). While testing juveniles require a substantial research effort within a short time frame, it is crucial to assess their learning abilities. This period may be critical for the establishment of cognitive functions, and numerous environmental factors can impact the development of learning abilities (Brust et al. 2014).

With our proposed test apparatus, we hope to provide interested researchers with a functional and efficient way to assess the associative learning performance suitable for different life stages of a large sample size. We believe that to gain a more comprehensive understanding of the animals in their natural conditions and the limitations of current findings, we should develop future research with wild populations in mind. Furthermore, we emphasise the need for better communication among researchers to bridge the gap and develop standardised methodologies for studying different cognitive domains. Streamlining the current procedures will also help future scientists who wish to implement these methods in the studies of other species and broaden our knowledge. Overall, our review provides a basis for future cognitive research in zebra finches and emphasises the need for continued exploration and collaboration in this field.

## Supplementary Information

Below is the link to the electronic supplementary material.

Supplementary file1 (XLSX 41 KB)

## Data Availability

Data has been included in the supplementary materials file with both the empirical and systematic review dataset.
